# Transmission and Control of *Plasmodium knowlesi:* A Mathematical Modelling Study

**DOI:** 10.1371/journal.pntd.0002978

**Published:** 2014-07-24

**Authors:** Natsuko Imai, Michael T. White, Azra C. Ghani, Chris J. Drakeley

**Affiliations:** 1 Department of Immunology and Infection, London School of Hygiene and Tropical Medicine, London, United Kingdom; 2 MRC Centre for Outbreak Analysis and Modelling, Department of Infectious Disease Epidemiology, Imperial College London, London, United Kingdom; Imperial College London, United Kingdom

## Abstract

**Introduction:**

*Plasmodium knowlesi* is now recognised as a leading cause of malaria in Malaysia. As humans come into increasing contact with the reservoir host (long-tailed macaques) as a consequence of deforestation, assessing the potential for a shift from zoonotic to sustained *P. knowlesi* transmission between humans is critical.

**Methods:**

A multi-host, multi-site transmission model was developed, taking into account the three areas (forest, farm, and village) where transmission is thought to occur. Latin hypercube sampling of model parameters was used to identify parameter sets consistent with possible prevalence in macaques and humans inferred from observed data. We then explore the consequences of increasing human-macaque contact in the farm, the likely impact of rapid treatment, and the use of long-lasting insecticide-treated nets (LLINs) in preventing wider spread of this emerging infection.

**Results:**

Identified model parameters were consistent with transmission being sustained by the macaques with spill over infections into the human population and with high overall basic reproduction numbers (up to 2267). The extent to which macaques forage in the farms had a non-linear relationship with human infection prevalence, the highest prevalence occurring when macaques forage in the farms but return frequently to the forest where they experience higher contact with vectors and hence sustain transmission. Only one of 1,046 parameter sets was consistent with sustained human-to-human transmission in the absence of macaques, although with a low human reproduction number (R_0H_ = 1.04). Simulations showed LLINs and rapid treatment provide personal protection to humans with maximal estimated reductions in human prevalence of 42% and 95%, respectively.

**Conclusion:**

This model simulates conditions where *P. knowlesi* transmission may occur and the potential impact of control measures. Predictions suggest that conventional control measures are sufficient at reducing the risk of infection in humans, but they must be actively implemented if *P. knowlesi* is to be controlled.

## Author Summary

*Plasmodium knowlesi* is a malaria of macaques which is now recognised as a leading cause of human malaria in Malaysia. Although current human infections are a result of human-macaque contact, there is a potential for *P. knowlesi* to be transmitted solely among humans. The authors developed a multi-host, multi-site transmission model to assess the likelihood of this happening due to increased human-macaque contact as a consequence of deforestation, population growth, and land-use change. How effective currently available malaria control measures were against *P. knowlesi* was also an important issue that was explored using the model. Although the model predicts that conventional control measures will be sufficient against *P. knowlesi*, with the push to eliminate malaria by the end of 2015, it is crucial to be aware of zoonotic malarias which may undermine such efforts.

## Introduction

Despite advances in the control and treatment of malaria more than half the world's population remain at risk of infection and disease. Of the estimated 216 million episodes of disease occurring worldwide in 2010, 13% were estimated to occur in South-East Asia [1]. In 2004, large numbers of malaria cases previously diagnosed as *Plasmodium malariae* in the Malaysian Borneo were discovered to be due to the simian *Plasmodium knowlesi* malaria [2]. *P. knowlesi* is a zoonotic malaria of macaques transmitted by the *Anopheles leucosphyrus* group of mosquitoes in South East Asia, and is increasingly recognised as a human malaria as incidence among humans continues to increase [3], [4]. It is difficult to determine whether the increase in reported *P. knowlesi* cases is genuine or a product of previous misdiagnosis as *P. malariae*. However the significant increase in the total number and proportion of malaria patients aged 50 years and above, an age group over-represented among genuine *P. knowlesi* patients suggests that there has been a true increase in *P. knowlesi* cases, especially as this coincides with reduced transmission of *P. falciparum* and *P. vivax* [4]. Although only recently confirmed in Malaysian Borneo, there is further evidence that *P. knowlesi* is much more widespread than previously thought with sporadic cases reported in China, Thailand, Myanmar and other neighbouring countries [5]–[9].

As an emerging infection that could become a major public health threat, it is critical to understand the extent to which transmission is maintained by the simian host population and whether wider spread of infections outside the traditional forested areas is likely. To date, the identified human *P. knowlesi* cases are mostly reported from individuals who have a history of exposure through proximity or travel to forest environments [10], supporting the premise that *P. knowlesi* is primarily zoonotic, with incidental human infections when humans encroach on non-human primate habitats at the forest-fringe. In addition, although *Anopheles latens* (a member of the *An. leucosphyrus* group and one of the main vectors in Malaysia) will feed on both non-human primates (NHPs) and humans, it is primarily a forest-feeder, and macaques found in human settlements have a lower prevalence of infection compared to their wild counterparts [5].

*P. knowlesi* was not considered an important cause of human malaria in the 1960s when vectors were typically found in the primary forest which covered much of Malaysia. However, with the extensive population growth of the last decades, humans encroach on large expanses of natural *P. knowlesi* transmission causing further habitat disruption and destruction. In response, NHPs have moved towards the forest fringes and mosquito vectors are increasingly found in human habitats [11]. Therefore, the increasing overlap between macaque, human, and vector habitats may in part explain the recent rise in *P. knowlesi* cases as humans are increasingly exposed to the vector and host, together increasing the probability of successful cross-species transmission. As an increasing number of *P. knowlesi* cases are reported from traditionally malaria-free areas, and with the push to eliminate malaria by the end of 2015, it is crucial to be aware of zoonotic malarias which may undermine such efforts. As such there is an urgent need to investigate appropriate treatment and prevention strategies [4], [11].

Here we develop a mathematical model for the transmission cycle of *P. knowlesi* incorporating the human, macaque and vector hosts allowing for human-vector-human transmission which has been demonstrated under laboratory conditions [12]. Although human-vector-human transmission has yet to be definitively documented in the wild, autochthonous cases reported in the Philippines [13], and the familial clustering of cases reported from a wide age distribution in Sabah, Malaysia are suggestive of peri-domestic transmission and point toward potential human-mosquito-human transmission [14]. Using parameters derived from the literature we estimate the extent to which infection is sustained by the different host populations and hence the potential for a shift from zoonotic to sustained transmission in the human population if human-macaque contact increases as a consequence of deforestation. We then use the model to assess the likely impact of rapid treatment and the use of insecticide-treated bed nets in preventing wider spread of this emerging infection.

## Methods

### Transmission Model

**Figure 1:**
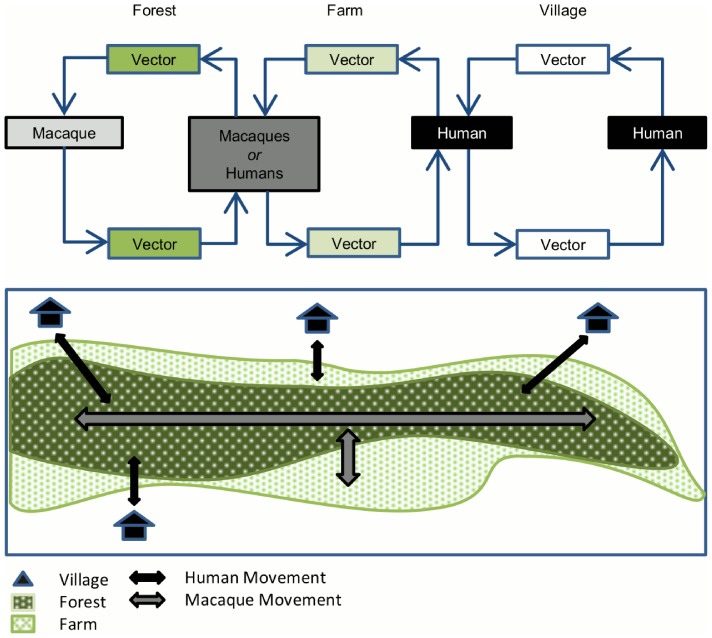
Distribution of forest, farm, villages, and the movement of macaques and humans between the 3 areas, and the hypothesised transmission cycle of *Plasmodium knowlesi* in Malaysia and Southeast Asia. Adapted from the yellow fever transmission cycle observed in Africa [52]. For clarity in notation, the forest will be referred to as the jungle (*J*), to differentiate from the farm (*F*) in later equations and model descriptions.

We extended a previous multi-host model for *P. knowlesi* transmission, which incorporated transmission between macaques, mosquitoes and humans [15], and then extended this further by accounting for three characteristic geographical sites (forest (J), farm (F) and village (V)) in which exposure to infection and transmission can occur. Here we define the forest as dense rainforest where macaques primarily reside, the farm area as an area on the forest-fringe that has been cleared for agricultural use where workers are present during the day, and the village as very small rural communities where humans live. A schematic of the model is shown in Figure 1 and full mathematical details are provided in the Supporting Information. Humans and macaques are assumed to move between locations (village-farm-forest and farm-forest respectively) whilst the vector population is stratified by each location. Each host (human, macaque, and vector) can be in one of two states – susceptible or infected – and hence we track the proportion of infected humans (*I_H_*), infected macaques (*I_M_*), and infected vectors in the forest (*I_VJ_*), farm (*I_VF_*) and village (*I_VV_*). A recovered compartment was excluded for macaques since infection is chronic, and for humans since data on immunity against *P. knowlesi* are not available.

We assume that humans and macaques are immediately infected and infectious following a mosquito bite [16], [17]. Parasitaemia peaks at day 8 after infection in humans, and falls rapidly to low levels by day 13 after infection. Thus we assumed a recovery rate of 1/14 days for humans [17]. We allow a delay in the vector transition from susceptible to infected state of 10 days to represent the extrinsic incubation period of the parasite [18]. Humans become infected at a rate λ_H_ which depends on (i) the rate at which vectors blood feed (accounting for human and macaque population sizes, the biting preference of the vector, and a reduction in biting rates on the farm to account for the absence of both humans and macaques in the evening when mosquitoes are most active [19]); (ii) the probability of transmission from mosquito to human/macaque per infectious bite; and (iii) the proportion of vectors infected in each location. Similarly vectors become infected in each of the three locations (forest, farm and village) at rates λ_VJ_, λ_VF_ and λ_VV_ respectively, which depend on their frequency of biting, the proportion of bites in each location taken on humans versus macaques and the prevalence of infection in humans or macaques. Since our model is not temporal, we have used the median vector biting rates from the literature [19]–[23]. Table 1 contains details of the parameters and values used and additional parameter values are given in the Supplementary Material (Table S1). To assess the potential for sustained transmission in the absence of the macaque population we also calculate the component of the basic reproduction number for a human-vector system, R_0H_. Full details are given in the Supporting Information.

**Table 1:** Parameters describing interactions between humans, macaques and mosquitoes derived from the literature.

Description	Symbol	Range (Value)	Ref
Human Density	Forest: 5%	Informed by census data chosen to equal average population density in the 3 areas as described in the methods.	[24]
	Farm: 30%		
	Village: 65%		
Macaque Density	Forest: 60%	16–60 macaques *km^−2^*	[53]–[55]
	Farm: 40%	29–87 macaques *km^−2^*	
	Village: 0%	0 macaques *km^−2^*	
Human Biting Rate	Forest	0–7 (1.24) *day^−1^*	[19]–[23]
	Farm	0–13.5 (1.11) *day^−1^*	
	Village	0–4.5 (1.11) *day^−1^*	
Vector Host Preference*	Forest: *Q_J_*	0.5	[53]–[55]
	Farm: *Q_F_*	0.5	
	Village: *Q_V_*	1	
Recovery Rate	r_H_: 1/14	1/(11–16) days	[17]
	r_Treated_: 1/5	-	Assumption
	r_M_: 1/2132	1/(368–3643) days	[56]
Mortality Rate	Human: *μ_H_*	1/29200 days	[24]
	Macaque: *μ_M_*	1/3650 days	[53]–[55]
	Vector: *μ_VJ_, μ_VF_, μ_VV_*	0.15 days	[15]
Extrinsic Incubation Period	*T*	10	[16], [17]
Transmission Co-efficient	Vector-Human: C_VH_	No comprehensive data available. Sensitivity analysis to help inform plausible values.	NA
	Vector-Macaque: C_VM_		
	Human-Vector: C_HV_		
	Macaque-Vector: C_MV_		
Gonotrophic cycle (day)	*δ*	3	[17], [19]
Frequency of biting in the absence of LLINs/LLIHs	*f_0_*	1/*δ*	
Time spent by mosquito searching for a blood meal (days)	T_1_	0.69	[32]
Time spent resting and ovipositing (days)	T_2_	*δ*−T_1_	
Proportion of encounters between mosquito and LLIN/LLIH protected human where net in use	φ_J_	*0.79*	[19] **
	φ_F_	*0*	
	φ_V_	*0.79*	

*(1 = human, 0 = macaque),

** Informed from % of bites after 8pm.

The total human population was fixed and distributed with 5%, 30% and 65% for the forest, farm, and village respectively. These percentages were chosen to reflect the proportion of time that an individual might spend in each location since our model does not explicitly include human movement. These values were chosen based on the Malaysian National Census and the average population density in corresponding areas [24]. Despite spending most of their time in the forest, macaques have been observed to encroach on farm land whilst foraging for food. The proportion of the total macaque population in daily contact with the farm was unknown. Furthermore there were very limited data available to inform the transmission probabilities between vectors and macaques, and vectors and humans, in addition to the infectious period among macaques.

In order to account for uncertainty in the parameters describing transmission of *P. knowlesi* between humans, macaques, and mosquitoes, and the unknown duration of infection in macaques, we undertook a model validation step using Latin hypercube sampling to obtain sets of these unknown parameters that were consistent with the possible prevalence of infection in humans and that in macaques. Due to its zoonotic nature, infection prevalence of *Plasmodium knowlesi* in humans in South East Asia is very low with an estimated annual incidence of 1% (95% CI: 0.4–1.7%) in southern Vietnam [25], 0.3% in Cambodia [26], and 0.65% in Thailand. In contrast, macaque *P. knowlesi* infection prevalence in the wild is extremely high at over 90%. Vythilingam *et al.*, compared *P. knowlesi* infection among urban and forest macaques in Malaysia and found that, while urban macaques were infection free, forest macaques had a prevalence of 97% [27]. Tan *et al.*, also found a prevalence of 87% in Sarawak among long-tailed macaques [28]. Since there are still very limited data on the true burden of *P. knowlesi* infection in humans and given that *P. knowlesi* is now the leading cause of malaria in Malayisan Borneo accounting for 87% of malaria admissions in Sabah [29], we allowed the upper limit of human infections to be high to reflect the possible range of prevalence using molecular detection tools [30], [31]. We therefore chose target ranges of 0–5% prevalence in humans and 80–100% prevalence in macaques for model validation. 50,000 parameter sets were selected using Latin hypercube sampling, and only those sets that resulted in infection prevalence within the target ranges were retained.

### Interventions

We considered the potential impact of two interventions on transmission – the provision of long lasting insecticidal nets and hammocks (LLINs, LLIHs) and more rapid treatment of human infections. To incorporate the former we adapted an approach previously described for models of *P. falciparum* transmission [32], [33]. Full mathematical details are given in the Supporting Information. In brief, the presence of a net reduces the biting rate on humans by providing direct protection to the individuals using a net; has a repellency effect which acts to increase the proportion of bites taken on other hosts and to increase the gonotrophic cycle length due to additional time spent searching for blood meals; and finally increases mosquito mortality due to the killing effect of the insecticide. Importantly, under this model, LLINs/LLIHs will affect the vector populations in the forest, farm and village differently, as there will be different numbers of humans sleeping under nets in each setting.

We considered the impact that different levels of LLINs/LLIHs coverage and usage may have in reducing *P. knowlesi* infection in humans, the human reproductive number, and the basic reproduction number. LLIN/LLIH usage in the farm was set to 0 since there is no evidence of net usage in this area. We do however assume that insecticide-treated hammocks (LLIHs) can be used in the forest [34], [35]. As a baseline scenario coverage (defined as the proportion of individuals in the population who always sleep under a net) was set at 80% in both the village and the forest based on a study in peninsular Malaysia [19].

To explore the impact of rapid treatment (a recovery rate of 1/5 days as opposed to 1/14 days) we varied the coverage from 0–100% and assumed that this would result in clearance of the parasites and hence reduce the duration of infection in the human host. This would then prevent onward transmission among humans and have a knock-on effect on the basic reproduction number for human infections.

## Results

Of the 50,000 parameter sets tested, 1,046 were compatible with the specified boundaries of 0–5% and 80–100% infection prevalence in humans and macaques respectively (Table 2). All of these scenarios were consistent with duration of infection in macaques of at least 1 year. In addition, the transmission probability from vectors to humans (*C_VH_*) was less than 0.5 in these scenarios, a condition that was required to match the low prevalence of infection in humans. The uncertainty in the other transmission probabilities could not be reduced by these constraints. In further sensitivity analyses, the human infection prevalence was found to be most affected by transmission probabilities directly involving humans (human-vector or vector-human transmission coefficients) (Figure S2). If human transmission coefficients are low, macaque transmission coefficients can be relatively high (allowing sustained transmission with an overall R_0_>1) yet still result in infection prevalence of less than 5% among humans. As expected, R_0H_ which describes human infection events with a human origin was only dependent on the parameters describing transmission between humans and mosquitoes indicating that these are the key parameters that would facilitate a shift towards human-human transmission, and independent of macaque-mosquito transmission.

**Table 2:** The median value and ranges of parameter values for *P. knowlesi* transmission probability and the macaque infectious period that satisfy the criteria at each model validation step.

Number of Parameter Sets	50, 000	1, 046	1
Criteria	-	<5% Human*	<5% Human*
		>80% Macaque*	>80% Macaque*
			R_0H_>1

	Range	Median (Range)	Value
Mean duration of infection in macaques (days)	1–3653	2132 (368–3643)	3378
Transmission Coefficient:			
Vector – Human (*C_VH_*)	0–1	0.01 (2×10^−4^–0.43)	0.14
Human – Vector (*C_HV_*)	0–1	0.45 (8×10^−5^–1.00)	0.62
Vector – Macaque (*C_VM_*)	0–1	0.53 (0.01–1.00)	0.60
Macaque – Vector (*C_MV_*)	0–1	0.27 (8.1×10^−4^–1.00)	0.001
R_0_	-	1.65–2267	2.0
R_0H_	-	1.0×10^−5^–1.04	1.04

*Infection prevalence.

All of the parameter sets were indicative of sustained macaque-to-macaque infection with human infections being driven by the high infection prevalence in the macaque population. Furthermore, of the 1,046 parameter sets that were consistent with the set target ranges, we only identified 1 scenario which was consistent with R_0H_>1 (Table 2). This scenario had R_0H_ and overall R_0_ values of 1.04 and 2.0 respectively. This scenario had a high vector-to-human and human-to-vector transmission probability (C_VH_ = 0.14, C_HV_ = 0.62 respectively) but a very small macaque-to-vector transmission probability (C_MV_ = 0.001). This single scenario where R_0H_ was greater than one had extreme values and such a low C_MV_ is highly unlikely given the high infection prevalence observed in macaques. This suggests that sustained human-human transmission is possible but unlikely particularly in the absence of macaques. Macaques infect more than six times as many mosquitoes as humans indicating that the human contribution to the overall system is currently small.

### Human-Macaque Mixing

**Figure 2:**
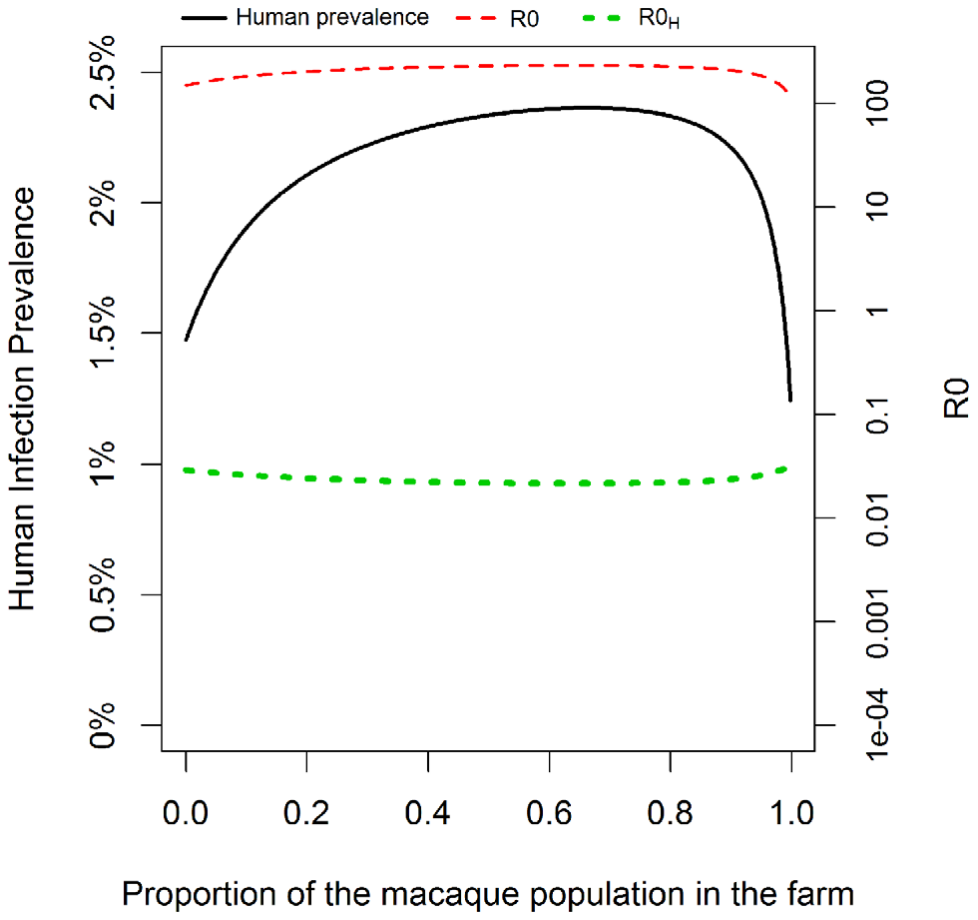
Change in total human infection prevalence, overall R_0_, and human R_0_ (R_0H_) with increased human-macaque mixing in the farm area.

Both human infection prevalence and the overall R_0_ depend on the proportion of macaques that spend time in the farm, with both these quantities reaching a peak when just over half of the macaque population are in the farm (Figure 2). When the majority of macaques remain in the forest, there is minimal overlap between areas where humans and macaques are active, and human infection prevalence stays low. As a greater proportion of the total macaque population are present in the farm, humans are increasingly infected. However when the ratio of macaques in the forest to farm reaches a certain threshold there is a switch to a situation of low infection prevalence in macaques as the high infection rates macaques experience in the forest are not maintained, and hence we observe a corresponding decrease in human prevalence.

### LLIN/LLIH Coverage

**Figure 3:**
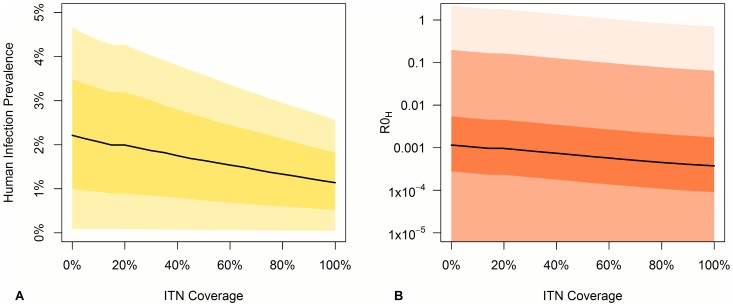
Change in a) total human infection prevalence and b) human R_0_ (R_0H_) with increasing LLIN/LLIH coverage in the village and jungle areas. Shaded areas represent for: (a) the 95% range (light yellow) and the interquartile range (dark yellow), (b) the full range (light pink) to enable us to see the coverage needed to bring R_0H_ below 1, the 95% range (light red), and the interquartile range (dark red). The block line represents the median value.

An increased LLIN/LLIH coverage in the village and forest is predicted to decrease human infection prevalence. At 100% coverage prevalence drops by approximately 40% due to the combined direct impact of personal protection and the indirect impacts of vector killing, repellency, and a longer gonotrophic cycle (Figure 3a). The low infection prevalence still observed with some plausible parameter sets at 100% coverage is due to infection in the farm, where LLINs/LLIHs are not assumed to be used, and also the small proportion of individuals not using a net even when they are available. The human component of the reproduction number under control (R_0H_C_) decreases as expected with increasing LLIN/LLIH coverage (Figure 3b), with coverage greater than 5% required to reduce R_0H_C_ to less than 1 in the single scenario in which R_0H_ was greater than 1 (represented by the pale pink area in Figure 3b and as described in Table 2).

### Rapid Treatment

**Figure 4:**
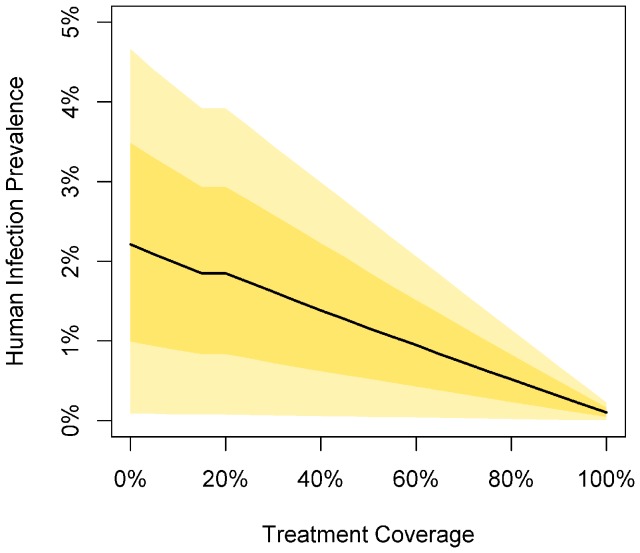
Change in total human infection prevalence with increasing rapid treatment coverage. Shaded areas represent the 95% range (light yellow) and interquartile range (dark yellow); the block line represents the median value.

As expected, human infection prevalence decreases rapidly with increasing coverage of access to rapid treatment where the infectious period in humans is three times shorter than when rapid treatment is not available (Figure 4). Since rapid treatment decreases the infectious period in humans, clearance of the parasite brings overall human infection prevalence down. Additionally, if all infected individuals were treated promptly, there would be minimal onward transmission from humans to vectors and hence a lower risk of infection from vectors to humans in the villages and farms.

## Discussion

Our model examines how *P. knowlesi* transmission may depend on different mixing patterns between humans and the primary host, long-tailed macaques, in different ecological settings. The model suggests that macaques sustain *P. knowlesi* transmission with minimal or no self-sustaining transmission between humans and vectors in the absence of macaques. However there is potential for this to change if macaque mixing patterns change in the farm (at the forest-fringe) with the highest infection prevalence among humans occurring when macaques forage in the farms but return sufficiently frequently to the forest where they experience higher contact with vectors and hence sustain transmission.

The model suggests that the majority of transmission is sustained and driven by the macaque population. This result is supported by data that show that patients presenting at hospitals infected with *P. knowlesi* were mostly subsistence farmers whose work took them into the forest or plantations associated with forest on a regular basis, or individuals who travelled through at risk areas [36]. As such, among the population at risk, the majority of infections occur in men aged 20–29 years [10]. However, *P. knowlesi* is now the most common cause of malaria in Sabah, Malaysia and true numbers of human infections may be missed by passive case detection at facilities. Recent studies have shown that all ages and both sexes are susceptible to infection with cases also reported in Malaysian children [29], and Vietnamese children [37], [38]. Additionally, familial clustering of cases has been demonstrated indicating transmission is probably now occurring peri-domestically contrary to previous reports, and that this may be linked to deforestation and/or land-use change in these environments [14].

The vector species that have been implicated in the transmission of *P. knowlesi* are numerous and the dynamics of many of these are poorly understood [23], [39]. Therefore additional data on the exact vector species present in the different ecological zones; forest canopy, forest ground level, farm, and village and their respective bionomic data including extrinsic incubation periods could be used to improve the model. *P. knowlesi* has not yet been reported beyond the range of the *An. leucosphyrus* group which are predominantly forest mosquitoes, occasionally found at forest fringes and open areas where presumably incidental human infection occurs. Experimentally, however, the entire Leucosphyrus group, comprised of 20 species, can transmit *P. knowlesi* under favourable conditions [16]. Thus it is probable that the current restriction of *P. knowlesi* to a vector which prefers the forest fringe habitat rather than a completely anthropophilic one has limited the emergence of *P. knowlesi* as a fully human malaria parasite and public health threat [5]. The likelihood is that where multiple vectors exist, such as in the Malaysian Borneo, they occupy distinct environmental niches with mosquito trapping likely to be logistically demanding [40]. The extent to which variations in species-specific host blood meal choice and susceptibility to plasmodial infection influence transmission dynamics is not known.

Even if infection becomes more prevalent in the human population and the domestic environment, it is the individuals who spend time in proximity to areas where macaques are also active, the farm or forest, who will remain at most risk of zoonotic *P. knowlesi* infection. Thus control measures directed to these at-risk areas and populations would be beneficial as a whole. Our simulations showed that with 100% LLIN/LLIH coverage in the village and the forest, human infection prevalence can be reduced by up to 42%. Studies looking at the effectiveness of bed nets on *P. falciparum* have reported overall protective effectiveness of 17%–54% [41], [42]. We have assumed that insecticide-treated hammocks (LLIH) can be used in the forest and that they are as effective as LLINs [35]. Magris *et al.*, found that LLIHs could reduce parasitaemia by 83% among the Yamomami people in Southern Venezuela [43]. Other studies have found reduction in malaria prevalence was 1.6 times greater when LLIHs were included in the intervention, with a 46% (95% CI: 35–55%) reduction in biting rates against *Anopheles minimus* in forested villages in Cambodia [35], [44]. Since the majority of infection is maintained in the forest by macaques, individuals who frequent these at risk areas should be made aware of the risks and encouraged to use LLIHs and other preventative options such as repellents as an easy and effective method of protection. However we did not find any impact on macaque infection prevalence with the use of LLIHs in the forest. The use of bed nets in the village will also become increasingly beneficial if human-human transmission becomes more frequent.

We found that rapid treatment of infected individuals to be the most effective in reducing infection prevalence among humans with a 95% reduction if every case is treated quickly (or within 5 days in our model). *P. knowlesi* has a rapid 24 hour erythrocytic cycle, and can result in severe and fatal infections if diagnosis and treatment are not prompt [3], [45]. Current observations show that *P. knowlesi* patients with uncomplicated malaria respond well to standard schizonticidal drugs with good prognosis and recovery after administration, with no relapse as *P. knowlesi* does not form dormant liver stages [16], [46]. There is no evidence for chloroquine-resistant *P. knowlesi* and as such chloroquine represents an inexpensive and highly effective therapy for uncomplicated *P. knowlesi* infections [47]. Additionally, since the majority of transmission is sustained by macaques, treatment of humans would not exert any substantial drug pressure.

As demonstrated in the model validation step, there are wide ranges of parameter values that are consistent with our current understanding of *P. knowlesi* from the limited data available. The upper bound of the overall R_0_ of 2267 was due to the extreme values of macaque to vector (C_MV_) and vector to macaque (C_VM_) transmission probabilities of 0.97 and 0.98 respectively in combination with a 10 year infectious period in macaques. Without detailed bionomic studies and empiric quantification of the natural history of *P. knowlesi*, it is impossible to reduce the uncertainties surrounding these values. Furthermore the role of super-infection has not yet been documented but it is plausible to assume that infection in macaques is more dynamic than the chronic infection assumed here. Additionally there are several other limitations to the model structure. In conventional malaria models individuals will move from susceptible to a pre-infectious compartment to take into account the latent period, around 9 −12 days from experimental studies in humans [12], rather than straight to an ‘infected’ compartment as set up here. Although the vector populations have been set up to incorporate the extrinsic incubation period crucial to any malaria model, it is assumed that humans and macaques are infectious immediately upon infection. Experimental observations suggest that although *P. knowlesi* produces gametocytes in mammalian hosts more rapidly than *Plasmodium falciparum*, they still take approximately 48 hours to develop and mature [16], [17]. Thus the addition of a pre-infectious period for humans and macaques would make the model more robust.

This model has assumed a constant seasonality in terms of vector and macaque densities. In many settings seasonality is a key factor in malaria transmission intensity where rainfall influences vector breeding and density. Seasonality is evident in the peak of *P. knowlesi* notifications in June in Sabah, Malaysia [4]. Seasonal fluctuations in the abundance and availability of different food types in the forest and on farms will also affect the behavior and density of macaques that move between these areas to forage and roost, and therefore any cross-species transmission that occurs may depend on the time of year.

Finally this model has been constructed based on the conditions observed in Malaysia and particularly in Sabah; thus predictions derived here may not be applicable to *P. knowlesi* infections elsewhere. With *P. knowlesi* cases being reported from several countries throughout South East Asia including Thailand [48], Singapore [49], [50], Indonesia [6], Vietnam [37], Myanmar [51], Cambodia [26], and the Philippines [13], both environmental conditions, demographics, and vector species involved are likely to be considerably different.

In summary, our results show that sustained human-vector-human transmission is unlikely to be occurring at present. However, as environmental change continues, there is the potential for the prevalence of *P. knowlesi* to increase and to become a significant public health problem. Our results highlight the need for sustained control and awareness of this zoonotic malaria particularly as Malaysia enters the pre-elimination stage for other malaria species.

## Supporting Information

Figure S1Diagrammatic representation of the effect of insecticide treated nets (ITN) on mosquitoes. Here *q_J_* and 1−*q_J_* are the proportion of bites taken on humans and macaques, respectively, in the forest, 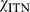 is ITN coverage in the area of interest, φ is the proportion of humans actually sleeping under an ITN, *p_1_* and *p_2_* are the probability that the mosquito survives the foraging and resting stage respectively, and T_1_ and T_2_ are the times spent in each category.(TIF)

Figure S2 A) Human infection prevalence with changing values of transmission efficacies, and B) Human R0 (R_0H_) with changing values of transmission efficacies. Parasite transmission efficacies are the product of their respective transmission coefficients. (Macaque-macaque parasite transmission efficacy = *C_MV_×C_VM_*, and human-human parasite transmission efficacy = *C_HV_×C_VH_*). The black contour line represents the 5% human infection prevalence and R0_H_ = 1 for figures A and B respectively.
(TIF)

Figure S3Change in relative biting rates (the proportion of bites taken on macaques (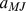) compared to humans (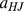)) in the jungle with ITN coverage.(TIF)

Table S1 Additional parameters and initial values.
(DOCX)

Text S1 Supplementary information containing full mathematical details, and further results from sensitivity analyses.
(PDF)
