# Supplementary material for: Transmission and Control of Plasmodium knowlesi: A Mathematical Modelling Study
Source: PLoS Negl Trop Dis. 2014 Jul 24;8(7):e2978. doi: 10.1371/journal.pntd.0002978 (PMC4109903; doi:10.1371/journal.pntd.0002978)
Supplement: Table S1 — Additional parameters and initial values. (DOCX) [file pntd.0002978.s004.docx]

**Table S1: Additional Parameters and Initial Values**

| Parameter Name | Symbol | (Initial) Value or Equations |
| --- | --- | --- |
| Proportion of Infected Humans | *I_H_* | 0 |
| Proportion of Infected Macaques | *I_M_* | 0.97 |
| Proportion of Infected Forest Vectors | *I_VJ_* | 0.039 |
| Proportion of Infected Farm Vectors | *I_VF_* | 0.027 |
| Proportion of Infected Village Vectors | *I_VV_* | 0.0026 |
| Number of vectors in the Forest | *N_VJ_* | $(N_{HJ}\times a_{HJ})/(f_{J}\times q_{J})$ |
| Number of vectors in the Farm | *N_VF_* | $(N_{HF}\times a_{HF})/(f_{F}\times q_{F})$ |
| Number of Vectors in the village | *N_VV_* | $(N_{HV}\times a_{HV})/(f_{V}\times q_{V})$ |
