# Supplementary material for: Transmission and Control of Plasmodium knowlesi: A Mathematical Modelling Study
Source: PLoS Negl Trop Dis. 2014 Jul 24;8(7):e2978. doi: 10.1371/journal.pntd.0002978 (PMC4109903; doi:10.1371/journal.pntd.0002978)
Supplement: Text S1 — Supplementary information containing full mathematical details, and further results from sensitivity analyses. (PDF) [file pntd.0002978.s005.pdf]

Transmission and control of *Plasmodium knowlesi:* a mathematical modelling study – Supporting Information

Natsuko Imai[1, 2], Michael T White[2], Azra C Ghani[2], Chris J Drakeley[1]
1 Department of Immunology and Infection, London School of Hygiene and Tropical Medicine, London, United Kingdom, **2** MRC Centre for Outbreak Analysis and Modelling, Department of Infectious Disease Epidemiology, Imperial College London, London, United Kingdom

Transmission Model

Transmission of *Plasmodium knowlesi* can be described by the following set of differential equations for the proportions of infected humans (I_H), infected macaques (I_M), and infected vectors in the forest (I_{VJ}), farm (I_{VF}) and village (I_{VV}):

$$\frac{dI_H}{dt} = \lambda_H (1 - I_H) - r_H I_H - \mu_H I_H$$ Infected humans

$$\frac{dI_M}{dt} = \lambda_M (1 - I_M) - r_M I_M - \mu_M I_M$$ Infected macaques

$$\frac{dI_{VJ}}{dt} = \lambda_{VJ} (e^{-\mu_{VJ} T} - I_{VJ}) - \mu_{VJ} I_{VJ}$$ Infected vectors – forest

$$\frac{dI_{VF}}{dt} = \lambda_{VF} (e^{-\mu_{VF} T} - I_{VF}) - \mu_{VF} I_{VF}$$ Infected vectors – farm

$$\frac{dI_{VV}}{dt} = \lambda_{VV} (e^{-\mu_{VV} T} - I_{VV}) - \mu_{VV} I_{VV}$$ Infected vectors – village

where r_H is the human recovery rate, μ_H is the human death rate, r_M is the macaque recovery rate, μ_M is the macaque death rate, μ_{VJ}, μ_{VF} and μ_{VV} are the death rates of mosquitoes in the forest, farm and village respectively, and T is the duration of sporogony.

The forces of infections in humans (λ_H) and macaques (λ_M) are given by

$$\lambda_H = \left(\frac{N_{VJ}}{N_{HJ}} a_{HJ} C_{VH} I_{VJ} \right) + \left(\frac{N_{VF}}{N_{HF}} a_{HF} C_{VH} I_{VF} \right) + \left(\frac{N_{VV}}{N_{HV}} a_{HV} C_{VH} I_{VV} \right)$$

$$\lambda_M = \left(\frac{N_{VJ}}{N_{MJ}} a_{MJ} C_{VM} I_{VJ} \right) + \left(\frac{N_{VF}}{N_{MF}} a_{MF} C_{VM} I_{VF} \right)$$

where N_{VJ}, N_{HJ} and N_{MJ} are the numbers of vectors, humans and macaques in the forest, a_{HJ} is the rate at which vectors in the forest bite on humans, and C_{VH} is the probability of transmission from mosquito to human per infectious bite. Other parameters are similarly defined.

The forces of infection on vectors in the forest (λ_{VJ}), farm (λ_{VF}) and village (λ_{VV}) are given by:

$$\lambda_{VJ} = a_{HJ} C_{HV} I_H + a_{MJ} C_{MV} I_M \quad \text{forest}$$

$$\lambda_{VF} = a_{HF} C_{HV} I_H + a_{MF} C_{MV} I_M \quad \text{farm}$$

$$\lambda_{VV} = a_{HV} C_{HV} I_H \quad \text{village}$$

The rate at which a mosquito bites humans or macaques depends on the frequency of biting (also equal to the inverse of the gonotrophic cycle duration) and the proportion of bites taken on humans or macaques:

$a_{HJ} = f_J q_J$ vectors on humans in the forest

$a_{MJ} = f_J (1 - q_J)$ vectors on macaques in the forest

$a_{HF} = f_F q_F$ vectors on humans in the farms

$a_{MF} = f_F (1 - q_F)$ vectors on macaques in the farms

$a_{HV} = f_V q_V$ vectors on humans in the village

$a_{MV} = f_V (1 - q_V)$ vectors on macaques in the village

where $f_J = f_F = f_V = f_0$ is the frequency of biting in the absence of ITNs, and q_J and 1 – q_J are the proportion of bites taken on humans and macaques, respectively, in the forest. The proportion of bites taken on humans and macaques will depend on their

relative densities and the preference for vectors on different hosts at the same densities (human = 1, macaque = 0):

$Q_J = 0.5$ in the forest

$Q_F = 0.5$ in the farm

$Q_V = 1$ in the village

Given the relative population sizes of humans and macaques the proportion of bites taken on humans can be calculated as:

$$q_J = \frac{N_{HJ} Q_J}{N_{HJ} Q_J + (1 - Q_J) N_{MJ}} \quad \text{in the forest}$$

$$q_F = \frac{N_{HF} Q_F}{N_{HF} Q_F + (1 - Q_F) N_{MF}} \quad \text{in the farm}$$

$$q_V = \frac{N_{HV} Q_V}{N_{HV} Q_V + (1 - Q_V) N_{MV}} \quad \text{in the village}$$

<u>Insecticide Treated Net (ITN) model</u>

The addition of insecticide treated nets will cause the following changes:

i. Reduced biting rate on protected humans
ii. Increased proportion of bites taken on macaques
iii. Increased mosquito mortality
iv. Increased gonotrophic cycle length due to additional time spent searching for blood meals

Importantly, ITNs will affect the vector populations in the forest, farm and village differently, as there will be different numbers of humans sleeping under nets in each region.

The equations detailed below are for vectors in the forest. Expressions for vectors in the farm and village can be derived analogously.

A schematic model showing the effect of ITNs on mosquitoes is shown in figure S2. Two key quantities are: w_J the probability that a surviving mosquito succeeds in feeding during a single attempt, and z_J the probability of a mosquito repeating and beginning a new search. These can be calculated as follows:

$$w_J = 1 - q_J + q_J(1-\chi_{\text{ITN}}) + q_J\chi_{\text{ITN}}(1-\varphi_J) + q_J\chi_{\text{ITN}}\varphi_J s_{\text{ITN}} = 1 - q_J\chi_{\text{ITN}}\varphi_J(1-s_{\text{ITN}})$$
$$z_J = q_J\chi_{\text{ITN}}\varphi_J r_{\text{ITN}}$$

Where q_J is the proportion of encounters between mosquito and LLIN/LLIH protected human where nets are in use, s_{ITN} is the proportion of encounters between mosquito and protected human that ends in a successful bite, and r_{ITN} is the proportion of encounters where the mosquito is repelled and must repeat its search for a blood meal.

First we calculate the additional time spent searching for a blood meal at ITN coverage χ_{ITN}. At zero ITN coverage, the duration of the gonotrophic cycle is given by:

$$\frac{1}{f_J} = \tau_1(0) + \tau_2$$

Increased ITN coverage will cause the mosquito to spend a longer time foraging for a blood meal. The time spent resting and ovipositing will remain the same. And hence at ITN coverage χ_{ITN}:

$$\tau_1(\chi_{\text{ITN}}) = \tau_1(0) + z_J\tau_1(\chi_{\text{ITN}}) = \frac{\tau_1(0)}{1-z_J}$$

And therefore the length of a feeding cycle at coverage χ_{ITN} is:

$$\frac{1}{f(\chi_{\text{ITN}})} = \frac{\tau_1(0)}{1-z_J} + \tau_2$$

The feeding frequency of mosquitoes on blood sources (humans or macaques) in the presence of ITNs in the forest will then be given by:

$$f_J = \left(\frac{\tau_1(0)}{1 - z_J} + \tau_2 \right)^{-1}$$

Secondly, we calculate the increased death rate of mosquitoes due to contact with ITNs. In the absence of ITNs, the probability that a mosquito survives the foraging, and resting and ovipositing stages is given by

$p_1(0) = e^{-\mu_{VJ}\tau_1}$ probability survives foraging

$p_2 = e^{-\mu_{VJ}\tau_2}$ probability survives resting and ovipositing

Increased ITN coverage will reduce the probability that a mosquito survives the foraging stage:

$$p_1(\chi_{\text{ITN}}) = p_1(0)[w + z_J\, p_1(\chi_{\text{ITN}})] = \frac{p_1(0) w_J}{1 - z_J\, p_1(0)}$$

Thus the probability of a mosquito surviving one day is given by

$$p_J(\chi_{\text{ITN}}) = \left(p_1(\chi_{\text{ITN}}) p_2 \right)^{f_J(\chi_{\text{ITN}})} = \left(\frac{p_1(0) w_J\, p_2}{1 - z_J\, p_1(0)} \right)^{f_J(\chi_{\text{ITN}})}$$

The mosquito mortality can then be calculated as

$$\mu_{VJ}(\chi_{\text{ITN}}) = -\log p_J(\chi_{\text{ITN}})$$

Thirdly, increasing ITN coverage will increase the proportion of bites taken on macaques and decrease the proportion taken on humans. The probability that an infectious mosquito takes a bite on a macaque is:

$$Z_M(\chi_{\text{ITN}}) = p_1(0)\left(1 - q_J + q_J\, \chi_{\text{ITN}} \varphi_J r_{\text{ITN}} Z_M(\chi_{\text{ITN}})\right) = \frac{p_1(0)(1 - q_J)}{1 - p_1(0) q_J\, \chi_{\text{ITN}} \varphi_J r_{\text{ITN}}}$$

And the probability that an infectious mosquito takes a bite on a human is:

$$Z_H(\chi_{\text{ITN}}) = p_1(0)\left(q_J - q_J\chi_{\text{ITN}}\varphi_J(1-s_{\text{ITN}}) + q_J\chi_{ITN}\varphi_J r_{\text{ITN}} Z_H(\chi_{\text{ITN}})\right) = \frac{p_1(0)q_J\left(1-\chi_{\text{ITN}}\varphi_J(1-s_{\text{ITN}})\right)}{1-p_1(0)q_J\chi_{ITN}\varphi_J r_{\text{ITN}}}$$

The proportion of bites taken on humans at ITN coverage χ_{ITN} is then given by:

$$q_J(\chi_{\text{ITN}}) = \frac{Z_H(\chi_{\text{ITN}})}{Z_H(\chi_{\text{ITN}}) + Z_M(\chi_{\text{ITN}})} = \frac{q_J - q_J\chi_{\text{ITN}}\varphi_J(1-s_{\text{ITN}})}{1-q_J\chi_{\text{ITN}}\varphi_J(1-s_{\text{ITN}})}$$

In the presence of ITNs, the biting rates on humans and macaques then becomes

$$a_{HJ}(\chi_{\text{ITN}}) = f_J(\chi_{\text{ITN}})q_J(\chi_{\text{ITN}})$$ biting rate on humans

$$a_{MJ}(\chi_{\text{ITN}}) = f_J(\chi_{\text{ITN}})\left(1-q_J(\chi_{\text{ITN}})\right)$$ biting rate on macaques

In our model, we fixed ITN usage in the farm at zero to account for the fact that nets cannot be used in this area. Additionally, due to the availability of insecticide-treated hammocks, we believe that the ITN model is realistic in the forest environment. Values of φ were informed from the literature and were set as: 0.79 in the village and forest (1).

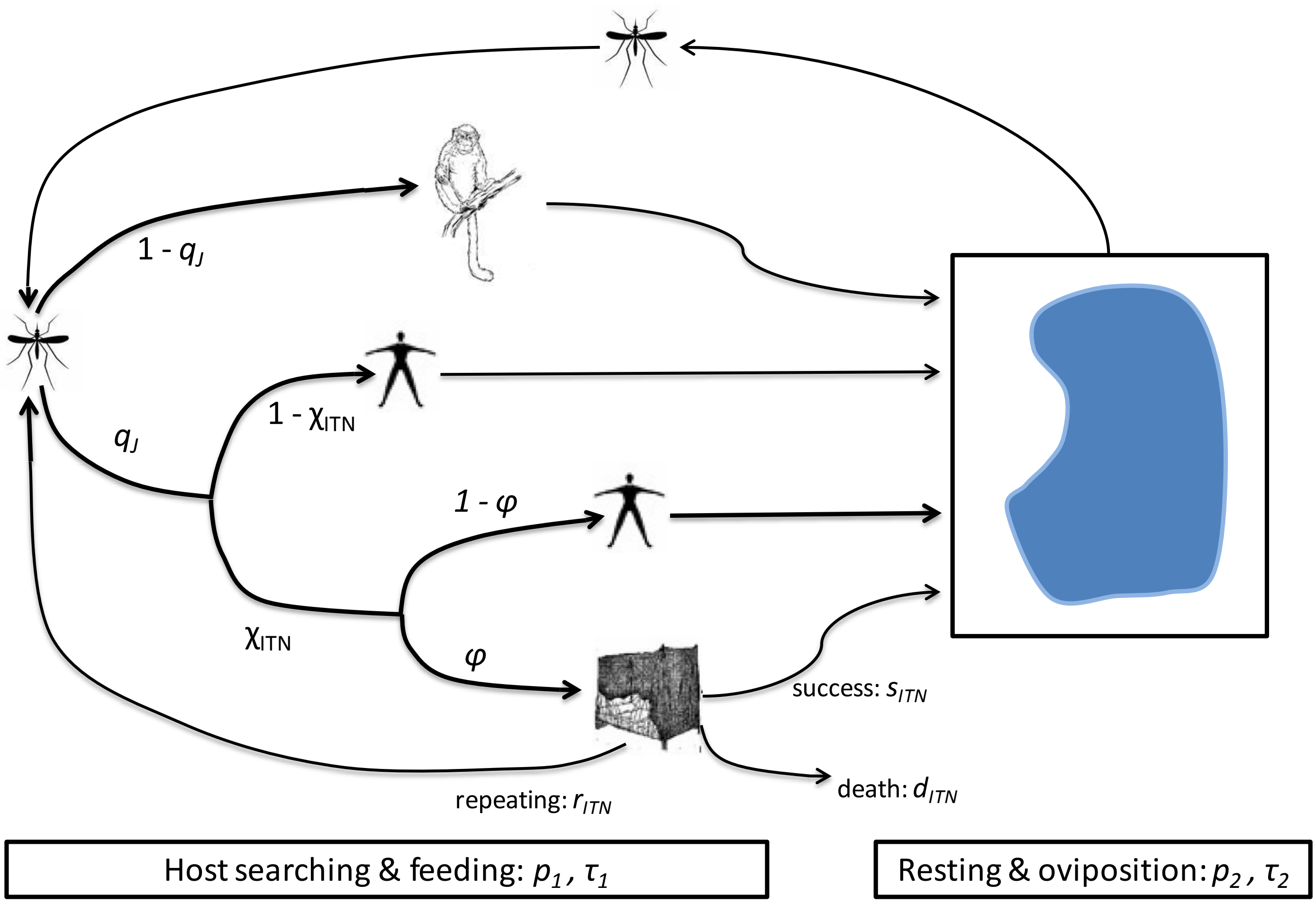

Figure S1: Diagrammatic representation of the effect of insecticide treated nets (ITN) on mosquitoes. Here q_J and $1 - q_J$ are the proportion of bites taken on humans and macaques, respectively, in the forest, χ_{ITN} is ITN coverage in the area of interest, φ is the proportion of humans actually sleeping under an ITN, p_1 and p_2 are the probability that the mosquito survives the foraging and resting stage respectively, and τ_1 and τ_2 are the times spent in each category.

Treatment Model:

Rapid treatment coverage is explicitly modelled by adding a ‘treated’ compartment which a fixed proportion of individuals join following the onset of infection. This can be modelled by the following equations:

$$\frac{dI_H}{dt} = (1 - T_{COV})\lambda_H (1 - I_H - I_T) - r_H I_H - \mu_H I_H$$ Infected humans

$$\frac{dI_T}{dt} = T_{COV}\lambda_H (1 - I_H - I_T) - r_{HTreated} I_H - \mu_{HTreated} I_H$$ Treated humans

where T_{COV} is the treatment coverage, I_T is the proportion of humans in the treated compartment, and $r_{HTreated}$ and $\mu_{HTreated}$ are recovery and mortality rates for treated individuals respectively. The equations for macaques and vector populations remain the same.

The Basic reproduction number, R_0

We follow the methodology outlined in van den Driessche and Watmough (2). Let $x = \left(I_H, I_M, I_{VJ}, I_{VF}, I_{VV}\right)$ be the vector of infected humans, macaques and vectors, and $x_0 = \left(0,0,0,0,0\right)$ be the disease free equilibrium. Let $\Upsilon_i(x)$ be the rate of appearance of new infections in compartment *i* and υ_i be the rate of transfer of individuals between compartments. Then the model of disease transmission can be written as

$$\dot{x}_i = \Upsilon_i(x) - \nu_i(x) \qquad i = 1,...,5$$

From van den Driessche and Watmough, if $F = \left[\frac{\partial \Upsilon_i}{\partial x_j}(x_0)\right]$ and $V = \left[\frac{\partial \upsilon_i}{\partial x_j}(x_0)\right]$ then $R_0 = \rho\left(FV^{-1}\right)$ where ρ is the spectral radius.

To make the notation a small bit easier, let $A_{JH} = \frac{N_{VJ}}{N_{HJ}} a_{HJ} C_{VH}$ and $A_{HJ} = a_{HJ} C_{HV} e^{-\mu_{VJ} T}$ and so forth. We first illustrate the calculation in the absence of ITNs where the simplification $\mu_{VJ} = \mu_{VF} = \mu_{VV} = \mu_V$ can be made. We calculate *F* and *V* as follows:

$$F = \begin{pmatrix} 0 & 0 & A_{JH} & A_{FH} & A_{VH} \\ 0 & 0 & A_{JM} & A_{FM} & 0 \\ A_{HJ} & A_{MJ} & 0 & 0 & 0 \\ A_{HF} & A_{MF} & 0 & 0 & 0 \\ A_{HV} & 0 & 0 & 0 & 0 \end{pmatrix} \text{ and } V = \begin{pmatrix} r_H + \mu_H & 0 & 0 & 0 & 0 \\ 0 & r_M + \mu_M & 0 & 0 & 0 \\ 0 & 0 & \mu_V & 0 & 0 \\ 0 & 0 & 0 & \mu_V & 0 \\ 0 & 0 & 0 & 0 & \mu_V \end{pmatrix}$$

We can then calculate $R_0 = \rho\left(FV^{-1}\right)$ to get:

$$R_0=\frac{1}{2}\left(\left(\frac{A_{HJ}A_{JH}}{(r_H+\mu_H)\mu_{VJ}}+\frac{A_{HF}A_{FH}}{(r_H+\mu_H)\mu_{VF}}+\frac{A_{HV}A_{VH}}{(r_H+\mu_H)\mu_{VV}}+\frac{A_{MJ}A_{JM}}{(r_M+\mu_M)\mu_{VJ}}+\frac{A_{MF}A_{FM}}{(r_M+\mu_M)\mu_{VF}}\right)+\sqrt{\begin{array}{l}\left(\frac{A_{HJ}A_{JH}}{(r_H+\mu_H)\mu_{VJ}}+\frac{A_{HF}A_{FH}}{(r_H+\mu_H)\mu_{VF}}+\frac{A_{HV}A_{VH}}{(r_H+\mu_H)\mu_{VV}}+\frac{A_{MJ}A_{JM}}{(r_M+\mu_M)\mu_{VJ}}+\frac{A_{MF}A_{FM}}{(r_M+\mu_M)\mu_{VF}}\right)^2-\\ \frac{4}{(r_H+\mu_H)(r_M+\mu_M)}\left(\begin{array}{l}\frac{A_{HV}A_{VH}A_{MJ}A_{JM}}{\mu_{VV}\mu_{VJ}}+\frac{A_{HV}A_{VH}A_{MF}A_{FM}}{\mu_{VV}\mu_{VF}}\\ +\frac{A_{HF}A_{FH}A_{MJ}A_{JM}}{\mu_{VF}\mu_{VJ}}+\frac{A_{HJ}A_{JH}A_{MF}A_{FM}}{\mu_{VJ}\mu_{VF}}\\ -\frac{A_{HJ}A_{FH}A_{MF}A_{JM}}{\mu_{VF}\mu_{VJ}}-\frac{A_{HF}A_{JH}A_{MJ}A_{FM}}{\mu_{VF}\mu_{VJ}}\end{array}\right)\end{array}}\right)$$

For R0 in the human population only, the expression simplifies to:

$$R_{0H}=\left(\frac{A_{HJ}A_{JH}}{(r_H+\mu_H)\mu_{VJ}}+\frac{A_{HF}A_{JF}}{(r_H+\mu_H)\mu_{VF}}+\frac{A_{HV}A_{JV}}{(r_H+\mu_H)\mu_{VV}}\right)$$

Table S1: Additional Parameters and Initial Values

Parameter Name	Symbol	(Initial) Value or Equations
Proportion of Infected Humans	I_H	0
Proportion of Infected Macaques	I_M	0.97
Proportion of Infected Forest Vectors	I_{VJ}	0.039
Proportion of Infected Farm Vectors	I_{VF}	0.027
Proportion of Infected Village Vectors	I_{VV}	0.0026
Number of vectors in the Forest	N_{VJ}	$(N_{HJ} \times a_{HJ})/(f_J \times q_J)$
Number of vectors in the Farm	N_{VF}	$(N_{HF} \times a_{HF})/(f_F \times q_F)$
Number of Vectors in the village	N_{VV}	$(N_{HV} \times a_{HV})/(f_V \times q_V)$

Supplementary Results

In a further sensitivity analysis, the impact of changing transmission efficacies (defined as the product of the respective transmission coefficients) on human infection prevalence (Figure S2a) and human R_0 (R_{0H}) (Figure S2b) were explored. Human infection prevalence and R_{0H} are most affected by transmission probabilities directly involving humans (human-vector or vector-human). If human transmission efficacy is low, macaque efficacy can be relatively high yet still result in low human infection prevalence and R_{0H}. As expected, R_{0H} was dependent on the parameters describing transmission between humans and mosquitoes, and independent of macaque-mosquito transmission.

With the introduction of LLINs/LLIHs in the forest the proportion of bites taken on macaques relative to humans increased as expected (Figure S3) as the LLIHs provide personal protection to individuals in the forest, the repelled mosquitoes are then diverted to an alternative blood host, in this case the macaques.

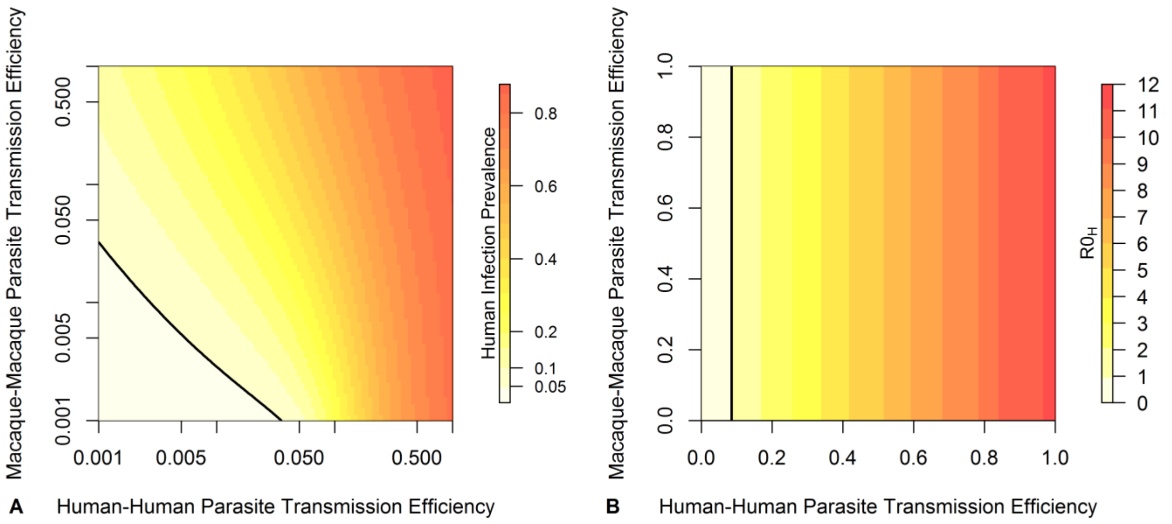

Figure S2: A) Human infection prevalence with changing values of transmission efficacies, and B) Human R0 (R_{0H}) with changing values of transmission efficacies. Parasite transmission efficacies are the product of their respective transmission coefficients. (Macaque-macaque parasite transmission efficacy = C_{MV} x C_{VM}, and human-human parasite transmission efficacy = C_{HV} x C_{VH}). The black contour line represents the 5% human infection prevalence and $R0_H$ = 1 for figures A and B respectively.

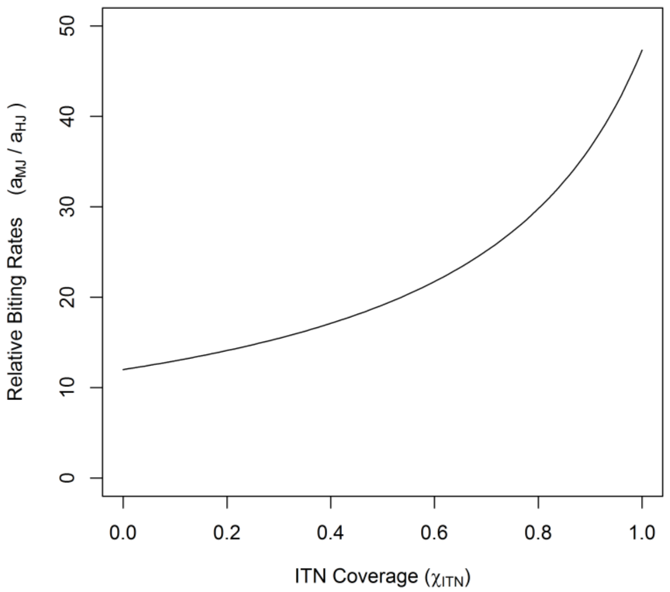

Figure S3: Change in relative biting rates (the proportion of bites taken on macaques (a_{MJ}) compared to humans (a_{HJ})) in the jungle with ITN coverage.

References

1. Jiram AI, Vythilingam I, Noorazian YM, Yusof YM, Azahari AH, Fong MY. Entomologic investigation of *Plasmodium knowlesi* vectors in Kuala lipis, Pahang, Malaysia. Malaria journal. 2012 Jun 22;11(1):213.
2. van den Driessche P, Watmough J. Reproduction numbers and sub-threshold endemic equilibria for compartmental models of disease transmission. Mathematical Biosciences. 2002 11//;180(1–2):29-48.
